# An overview of the European Health Examination Survey Pilot Joint Action

**DOI:** 10.1186/0778-7367-70-20

**Published:** 2012-08-28

**Authors:** Kari Kuulasmaa, Hanna Tolonen, Päivikki Koponen, Katri Kilpeläinen, Mária Avdicová, Grazyna Broda, Neville Calleja, Carlos Dias, Antje Gösswald, Ruzena Kubinova, Jennifer Mindell, Satu Männistö, Luigi Palmieri, Grethe S Tell, Antonia Trichopoulou, WM Monique Verschuren

**Affiliations:** 1 National Institute for Health and Welfare (THL), Helsinki, Finland; 2 Regional Authority of Public Health, Banská Bystrica, Slovakia; 3 The Cardinal Stefan Wyszynski Institute of Cardiology, Warsaw, Poland; 4 Ministry for Social Policy, Valletta, Malta; 5 Instituto Nacional de Saúde Dr. Ricardo Jorge, Lisbon, Portugal; 6 Robert Koch Institute, Berlin, Germany; 7 National Institute of Public Health, Prague, Czech Republic; 8 UCL (University College London), London, UK; 9 Istituto Superiore di Sanità, Rome, Italy; 10 Norwegian Institute of Public Health, Bergen, Norway; 11 Hellenic Health Foundation, Athens, Greece; 12 National Institute of Public Health and the Environment (RIVM), Bilthoven, Netherlands

**Keywords:** Health surveys, Population health monitoring, Risk factors, Chronic diseases, EHES, Survey methods

## Abstract

**Background:**

Health Examination Surveys (HESs) can provide essential information on the health and health determinants of a population, which is not available from other data sources. Nevertheless, only some European countries have systems of national HESs. A study conducted in 2006–2008 concluded that it is feasible to organize national HESs using standardized measurement procedures in nearly all EU countries. The feasibility study also outlined a structure for a European Health Examination Survey (EHES), which is a collaboration to organize standardized HESs in countries across Europe.

To facilitate setting up national surveys and to gain experience in applying the EHES methods in different cultures, EHES Joint Action (2010–2011) planned and piloted standardized HESs in the working age population in 12 countries. This included countries with earlier national HESs and countries which were planning their first national HES. The core measurements included in all surveys were weight, height, waist circumference and blood pressure, and blood samples were taken to measure lipid profiles and glucose or glycated haemoglobin (HbA_1c_). These are modifiable determinants of major chronic diseases not identified in health interview surveys. There was a questionnaire to complement the data on the examination measurements.

**Methods:**

Evaluation of the pilot surveys was based on review of national manuals and evaluation reports of survey organizers; observations and discussions of survey procedures during site visits and training seminars; and other communication with the survey organizers.

**Results:**

Despite unavoidable differences in the ways HESs are organized in the various countries, high quality and comparability of the data seems achievable. The biggest challenge in each country was obtaining high participation rate. Most of the pilot countries are now ready to start their full-size national HES, and six of them have already started.

**Conclusions:**

The EHES Pilot Project has set up the structure for obtaining comparable high quality health indicators on health and important modifiable risk factors of major non-communicable diseases from the European countries. The European Union is now in a key position to make this structure sustainable. The EHES core survey can be expanded to cover other measurements.

## Background

Administrative and disease-specific registers, questionnaire surveys and health examination surveys (HESs) are the main sources of population level information on the health and health related aspects of the residents of countries. HESs can provide objective information on many conditions, including those of which the person is unaware or which are not recorded systematically or in a comparable way in the health care system. Examples of such conditions are hypertension and type 2 diabetes. Each of these is an important risk factor for major but preventable chronic disability.

Some European countries and the United States of America have repeated national HESs [1-6], but in many countries such data are not available. Comparability between the existing data is hindered by lack of standardization. The WHO MONICA Project for monitoring trends and determinants of cardiovascular diseases standardized HESs in 21 countries, mostly from Europe [7]. However, MONICA ended in the late 1990s, and did not cover whole countries. WHO has developed a simple STEPS approach for risk factor monitoring, with focus on low and middle income countries [8].

There is a need for HES data from more European countries for the evidence base to support the planning and evaluation of health policies. Thus, a feasibility study of European HESs was conducted in 2006–2008 [9]. It concluded that it is feasible to carry out national HESs in nearly all European countries and found that 17 countries already had plans to start national HESs in the next five years. Therefore Europe-wide collaboration to standardize national HESs was needed immediately. At the same time, the health strategy for 2008–2013 of the Commission of the European Union called for collection of comparable health data [10]. The EU regulation on Community statistics on public health specified that all countries must carry out European Health Interview Surveys (EHIS) and the implementation of HESs is optional [11,12].

This brief communication describes how the European Health Examination Survey (EHES) was set up, with specific focus on the EHES Pilot Joint Action for planning a national HES and testing its organization and methods in twelve countries. An overview of the experiences from this Joint Action is provided. Details of the experiences on sampling, recruitment and the different EHES measurements will be reported separately.

### Structure of EHES

The feasibility study recommended a structure for EHES and a number of core measurements which should be included by all countries [9,13]. The national surveys should be organized and carried out by national experts. There should be a reference centre at EU level responsible for:

• the European level coordination,

• defining and maintaining European measurement standards,

• advising the countries on various aspects of planning and implementation of the surveys,

• organizing training and external quality assessment, and

• evaluation of the national HESs and undertaking basic reporting at the European level.

The target population would be the 25–64 years old residents of the whole countries. The surveys should use probability sampling, where every eligible individual or household has a known probability of being sampled. A sample size of 4000 persons per country would be sufficient for a meaningful precision of national indicators. It would also allow simple comparisons between population sub-groups, such as socio-economic classes. Depending on feasibility and national interests, the target population could be extended to all adults aged 18 and over.

The core measurements are weight, height, waist circumference, blood pressure, and blood samples for the measurement of lipid profile and fasting glucose or glycated haemoglobin (HbA_1c_) to assess type 2 diabetes. The core questionnaire provides additional information needed for proper interpretation of the measurement results, such as the level of education, and awareness and treatment of hypertension. The selection of core measurements was based on epidemiological and public health criteria, availability of international standards, and practicality for large population surveys [9]. Countries can add measurements based on national priorities, and availability of experience and funding. For example, it is possible to combine the HES with EHIS [12]. Countries with little experience with HESs are advised not to include many additional measurements.

### Setting up EHES

Following the recommendations of the feasibility study, EHES Pilot Project was included in the 2009 Work Plan of the EU Health Programme, and funded through a EU Service Contract and a Joint Action [14]. The EHES Reference Centre was funded for two years through the Service Contract and established jointly by the National Public Health Institutes of Finland and Italy, and Statistics Norway. EHES Pilot Joint Action was set up to plan and prepare for national HESs in the first 14 countries. The preparation included a pilot for fieldwork to collect data on 200 participants, data assessment and reporting. The Joint Action was coordinated by the National Institute for Health and Welfare of Finland [15].

Some of the pilot countries had no earlier national HESs. For them, the objective was to find out how to implement EHES in their country and to gain experience on the various steps of conducting a national HES. For the countries with earlier national HESs, the aim was to examine the extent to which they could synchronize their surveys with EHES without losing the ability to follow trends from their past surveys.

## Methods

The experience from the EHES Joint Action is based on a review of the national HES manuals prepared by countries; observation and discussion of the survey procedures during site visits conducted by the EHES Reference Centre personnel during the pilot survey field work; laboratory external quality assessment organized by the EHES Reference Centre; reports and discussions of the survey organizers during two EHES training seminars and an EHES Workshop on Cultural Adaptations; pilot survey evaluation reports prepared by the survey organizers; and other communication between the Joint Action partners and the EHES Reference Centre.

### Results of the Joint Action

**Figure 1:**
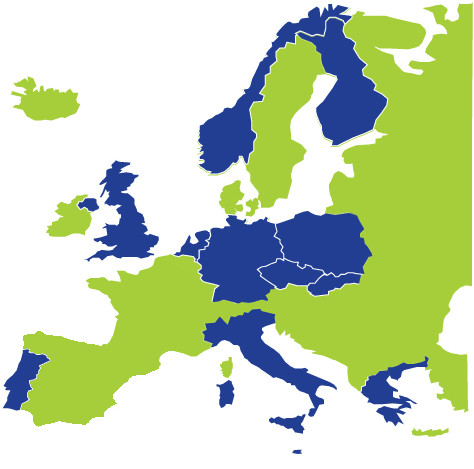
**Countries completing the EHES Joint Action.** The EHES Joint Action was completed by the Czech Republic, Finland, Germany, Greece, Italy, Malta, the Netherlands, Norway, Poland, Portugal, Slovakia and UK/England.

Preparation for the national HES was completed by 12 of the 14 countries (Figure 1). One country withdrew from the Joint Action at an early stage because of a change in priorities following a change of government. Another country withdrew towards the end of the Joint Action after failing to fulfil the commitments of the Joint Action. All other countries completed the Joint Action and are now technically prepared and confident to proceed to full-size national HESs. Four of the countries (Germany, Italy, The Netherlands, UK/England) started a full-size national HES before or at an early stage of the Joint Action. These incorporated the piloting activities in the full-size survey and examined implications of replacing their earlier methods with the EHES standards.

The European-level collaboration helped build capacity in the countries. The pilot surveys provided valuable information on conducting HESs in different settings and cultures. For example, personal contacts helped to improve participation, but in some countries they were difficult to implement because of incomplete contact information. Site visits by the EHES Reference Centre personnel during the pilot surveys revealed various shortcomings in the measurement environment and procedures, which could mostly be corrected for the rest of the field work. Differences in the examination methods between earlier and ongoing surveys and the EHES standards were generally small and did not compromise comparability. Standardizing the questionnaires was more problematic in some countries because a change to using the EHES questionnaire could have compromised the assessment of trends from previous surveys. The fact that the Health Survey for England was based on home visits while in the other countries the measurements were carried out during clinic visits led to difficulties in standardization, especially for the blood samples.

The survey organizers often do not have expertise in sampling. Therefore, support from the survey statisticians of the EHES Reference Centre at Statistics Norway was important. Good sampling frames were generally available, although they were not always up-to-date.

Apart from funding, the biggest challenge in each country is obtaining high participation rate. None of the pilot surveys reached the target participation rate of at least 70%.

## Discussion

The main objectives of EHES Pilot Joint Action were to plan and prepare for full-size surveys in the European countries actively planning or already carrying out national HESs. These objectives were met well in the twelve countries which completed the Joint Action. The Joint Action revealed the power of collaboration in the planning and preparation for surveys and of learning from the experiences of others. This was appreciated both by the countries which were planning their first national HES and those with existing periodic or annual HESs. The same is true regarding the training programme and external quality assessment. Support in sampling design was important to ensure that representative health indicators and their precision can be estimated accurately.

Germany, Italy, Netherlands and UK/England started their full-size HESs before or early during the Joint Action period. Slovakia conducted a full-size HES after the pilot survey at the end of year 2011, and Finland conducted one in the beginning of 2012. The other six piloting countries are expected to start in 2012–2014, depending on funding. In addition, Luxembourg is planning to start a national HES in 2012 and France in 2013. Although these did not participate in the Joint Action, they have collaborated with EHES for the standardization.

The pilot surveys are too small to provide precise estimates of health indicators, and represent only small areas of the countries. Therefore they cannot be used to infer information about health in these countries or across Europe, but they are important for testing and further developing the survey methods and their national adaptations. This paper was written soon after the end of the Joint Action. The assessment of the data from the pilot surveys is ongoing, and will provide more information on the quality of the pilot surveys.

The piloting countries have used the pilot survey data to test local reporting. DG Health and Consumers of the European Commission has prepared the HEIDI data tool for the European level reporting of health indicators [16]. The suitability of HEIDI for reporting the EHES data will be tested.

The EHES Pilot Project has set up a structure for EHES to provide high quality comparable health indicators on major public health issues which cannot be monitored in other ways. The structure consists of nationally conducted HESs and the EHES Reference Centre, to provide information to the HEIDI reporting system. Each country is responsible for conducting and primarily funding the national surveys. However, partial financial support from the EU would lower significantly the threshold for countries joining EHES. An EHES Reference Centre is needed to maintain the European standards; provide support to the countries, to ensure the comparability of the national data; and to facilitate joint European level reporting of the forthcoming full-size HESs. Hence it should be funded at the EU level. This funding is not currently available, but a recent conclusion adopted by the Council of the EU recognizes the importance of the sustainability of health monitoring [17].

Obtaining high participation rates is a major challenge in all population surveys. The EHES Joint Action tested various approaches in different cultures; further development of innovative approaches is needed. It is also likely that more resources will be required for participant recruitment in the future.

It is a prerequisite for EHES that the national and European regulations and principles on ethics and data protection recognize the role of HESs for the benefit of public health. In one piloting country, the national principles for limited contacts with the selected persons seriously restricted the efforts to obtain a high participation rate and therefore the ability to obtain representative information. The EU Data Protection Directive has now been opened for revision: we hope it will facilitate future public health monitoring and research in all countries [18].

In addition to data demands expressed in the EU’s policy statements when the pilot phase of EHES started, EHES can provide much of the key data called for in the Political Declaration of the United Nations High-level Meeting on Non-communicable Diseases in 2011 and in the WHO/Euro Action plan for the Strategy for the Prevention and Control of Non-communicable Diseases for 2012–2016 [11,14,19,20].

EHES will also be a unique data source for epidemiologic and public health research, with high potential to contribute to the objectives of the Europe 2020 Flagship Initiative Innovation Union [21]. The EHES Pilot Project is creating data sharing principles which will facilitate wide research use of the data while respecting the legitimate interests of the survey participants and organizers.

## Conclusions

There is wide recognition of the importance of HESs as a part of national health monitoring systems. The EHES Pilot Project has set up the structure for obtaining comparable high quality health indicators on health and important modifiable risk factors of major non-communicable diseases from the European countries. The European Union is now in a key position to make this structure sustainable. The EHES core survey can be expanded to cover other measurements.

## Abbreviations

EHES: European Health Examination Survey; EHIS: European Health Interview Survey; EU: European Union; HES: Health examination survey.

## Competing interests

The authors declare that they have no competing interests.

## Authors’ contributions

All authors participated in the design of the study and data preparation. KKu drafted the paper. HT was the Project Manager of the EHES Pilot Project and helped in drafting the manuscript. PK was responsible for the EHES Training programme and was involved in evaluating the pilot surveys and helped in drafting the manuscript. KKi coordinated the EHES Pilot Joint Action. MA, GB, NC, CD, AG, RK, JM, SM, LP, GT, AT and MV were responsible for the national adaptations of EHES and evaluation of the national pilot studies. All authors read and approved the final manuscript.

## Disclaimer

The EHES Pilot Project has received funding from the European Commission/Health and Consumers. The views expressed here are those of the authors and they do not represent Commission’s official position.

## Supplementary Material

Additional file 1 Sites and key personnel contributing to the EHES Pilot Project. Click here for file
