# Supplementary material for: An overview of the European Health Examination Survey Pilot Joint Action
Source: Arch Public Health. 2012 Aug 28;70(1):20. doi: 10.1186/0778-7367-70-20 (PMC3508610; doi:10.1186/0778-7367-70-20)
Supplement: Additional file 1 — Sites and key personnel contributing to the EHES Pilot Project. [file 0778-7367-70-20-S1.doc]

**Additional file 1: Sites and key personnel contributing to the EHES Pilot Project**

**Czech Republic**

National Institute of Public Health, Prague: Ruzena Kubinova, Nada Capkova, Jana Kratenova

and Michala Lustigova

**Finland**

National Institute for Health and Welfare (THL). EHES Reference Centre: Kari Kuulasmaa, Hanna Tolonen, Katri Kilpeläinen, Päivikki Koponen, Sanna Ahonen, Johanna Mäki-Opas, Ari Haukijärvi, Tarja Tuovinen, Georg Alfthan, Jari Kirsilä;

National pilot survey: Satu Männistö, Katja Borodulin, Liisa Saarikoski, Anne Juolevi, Markku Peltonen, Tiina Laatikainen, Erkki Vartiainen, Jouko Sundvall, Laura Lund, Antti Jula, Eija Purkamo.

**Germany**

Robert Koch Institute, Berlin. For the DEGS Study Team: Antje Gösswald, Cornelia Lange, Panagiotis Kamtsiuris.

**Greece**

Hellenic Health Foundation, Athens. Antonia Trichopoulou, Valentini Konstantinidou, Androniki Naska, Dimosthenis Zilis, Vardis Dilis, George Adarakis, Ioulia Goufa, Georgia Stasinopoulou, Elisabeth Valanou, Perikles Karathanasis, Nikolaos Bilalis, Philippos Orfanos, Tina Karapetyan, Despina Oikonomidou, Eirini Frangogeorgi and Konstantinos Mine.

**Italy**

Istituto Superiore di Sanità, Rome. EHES Reference Centre: Susanna Conti, Mark Kanieff;

National Pilot Survey: Luigi Palmieri, Chiara Donfrancesco, Cinzia Lo Noce, Francesco Dima, Amalia De Curtis, Licia Iacoviello, Diego Vanuzzo, Simona Giampaoli.

**Malta**

Department of Health Information & Research, Gwardamangia: Neville Calleja, Dorothy Gauci.

**The Netherlands**

National Institute of Public Health and the Environment (RIVM), Bilthoven: W.M.Monique Verschuren.

**Norway**

Norwegian Institute of Public Health: Grethe S. Tell, Patricia Schreuder, Sidsel Graff-Iversen, Nina Hovland;

University of Bergen: Kristin Klock;

Statistics Norway. EHES Reference Centre: Johan Heldal, Susie Jentoft.

**Poland**

The Cardinal Stefan Wyszynski Institute of Cardiology, Warsaw: Grażyna Broda, Aleksandra Piwonska, Jerzy

Piwoński, Paweł Kurjata, Walerian Piotrowski, Maria Polakowska, Anna

Waśkiewicz, Elzbieta Sygnowska.

**Portugal**

Instituto Nacional de Saúde Dr. Ricardo Jorge, Lisbon: Carlos Dias, Ana Paola Gil.

**Slovakia**

Regional Authority of Public Health, Banská Bystrica. Maria Avdicova, Katarina Francisciova, Jana Namesna, Silvia Kontrosova.

**UK**

UCL (University College London), London: Jennifer Mindell, Nicola Shelton, Barbara Carter-Szatynska, Alison Moody;

NatCen Social Research, London: Rachel Craig, Susan Nunn, Deanna Pickup, Chloe Robinson;

The NHS Information Centre: Steve Webster, Victoria Cooper.
